# OP15 Incorporating Lived Experiences In Health Technology Assessment: Impact On Subsidy Recommendations For Continuous Glucose Monitoring Systems In Singapore

**DOI:** 10.1017/S0266462325100731

**Published:** 2025-12-29

**Authors:** Ping-Tee Tan, Fiona Pearce, Shawn Quek

## Abstract

**Introduction:**

Since 2023, the Agency for Care Effectiveness (ACE) has included lived experiences from patients and caregivers in health technology assessments (HTAs) for wearable or home-based medical devices. This presentation discusses the impact of patient experience data on the HTA and subsequent subsidy recommendations for continuous glucose monitoring (CGM) systems for type 1 diabetes mellitus (T1DM) in Singapore.

**Methods:**

All local patient organizations with an interest in CGM systems or T1DM were invited to share their views using a structured, qualitative survey or simple free-text patient journey form. Testimonials were analyzed by ACE to extract key themes and insights and incorporated into the HTA report alongside clinicians’ input and clinical and economic evidence to inform subsidy recommendations made by the Ministry of Health Medical Technology Advisory Committee. The impact of patient experience data on the Committee’s deliberations and recommendations for CGM systems was determined by reviewing the HTA report, minutes from Committee meetings, and the published HTA guidance document.

**Results:**

Eighty testimonials were received from patients and caregivers about the impact T1DM has on their lives, and their experiences using different blood glucose monitors. They also confirmed real-world CGM usage across different age groups, informing assumptions for budget impact calculations. Testimonials highlighted benefits and drawbacks of different monitoring methods, unmet needs, patient preferences, and expectations for new technologies to be more affordable and less invasive. While scientific evidence for children with T1DM was limited, testimonials confirmed CGM systems could improve outcomes for both adults and children, which influenced the subsidy criteria recommended by the Committee to cover both age groups.

**Conclusions:**

Incorporating patients’ lived experiences in ACE’s HTAs validated and strengthened the scientific evidence, addressed uncertainties by filling data gaps, and led to patient-centered subsidy recommendations for CGM systems, demonstrating the value of systematic patient involvement. Post-recommendation, ACE co-developed a factsheet on CGM systems with local patient organizations to explain the subsidy recommendations in plain language and encourage HTA guidance adoption.

